# Infliction of proteotoxic stresses by impairment of the unfolded protein response or proteasomal inhibition as a therapeutic strategy for mast cell leukemia

**DOI:** 10.18632/oncotarget.23354

**Published:** 2017-12-17

**Authors:** Thomas Wilhelm, Fabian Bick, Kerstin Peters, Vrinda Mohta, Boaz Tirosh, John B. Patterson, Behzad Kharabi-Masouleh, Michael Huber

**Affiliations:** ^1^ Institute of Biochemistry and Molecular Immunology, Medical Faculty, RWTH Aachen University, Aachen, Germany; ^2^ The Institute of Drug Research, The Hebrew University of Jerusalem, Jerusalem, Israel; ^3^ Fosun Orinove, Inc., Newbury Park, CA, USA; ^4^ Department of Hematology, Oncology, Hemostaseology and Stem Cell Transplantation, Medical Faculty, RWTH Aachen University, Aachen, Germany

**Keywords:** IRE1α, proteasome, proteostasis, stress kinase, XBP1

## Abstract

The intensity and duration of endoplasmic reticulum (ER) stress converts the unfolded protein response (UPR) from an adaptive into a terminal response. The first regulates homeostasis, the latter triggers apoptosis. Cells that rapidly proliferate and possess developed secretory capabilities, such as leukemia cells, depend on an efficiently operating UPR to maintain proteostasis. Activation of terminal UPR by either blockade of adaptive UPR or exaggeration of ER stress has been explored as a novel approach in cancer therapy. For mast cell leukemia (MCL) the efficacy of both approaches, by utilizing the KIT^V560G,D816V^-positive MCL cell line HMC-1.2, was investigated. We show that HMC-1.2 cells display a tonic activation of the IRE1α arm of the UPR, which constitutively generates spliced XBP1. Inhibition of IRE1α by different types of inhibitors (MKC-8866, STF-083010, and KIRA6) suppressed proliferation at concentrations needed for blockade of IRE1α-mediated *XBP1* splicing. At higher concentrations, these inhibitors triggered an apoptotic response. Blocking the proteasome by bortezomib, which confers an exaggerated UPR, resulted in a marked cytotoxic response. Bortezomib treatment also caused activation of the kinase JNK, which played a pro-proliferative and anti-apoptotic role. Hence, the combination of bortezomib with a JNK inhibitor synergized to induce cell death. In summary, the UPR can be addressed as an effective therapeutic target against KIT^D816V^-positive MCL.

## INTRODUCTION

For physiological performance, cells depend on a functional proteostasis network enabling proper protein expression, folding, transport and topology, as well as the capability to stringently control and, when necessary, to correct these processes upon stressful perturbations [1]. The endoplasmic reticulum (ER) is the port of entry of proteins into the secretory pathway. Particularly in cells with a pronounced proliferative and/or secretory phenotype, such as cancer cells, extensive protein and membrane production as well as protein folding tasks are required. Hence, cancer cells exhibit vulnerability to the accumulation of misfolded proteins in the ER. An elaborate system comprising sensors, transcription factors and chaperones, called the unfolded protein response (UPR), has been evolutionary established to maintain proteostasis [2, 3]. Three transmembrane sensor proteins located in the ER membrane form the backbone of the UPR – inositol-requiring enzyme 1α (IRE1α), double-stranded RNA-activated protein kinase-like ER kinase (PERK), and activating transcription factor 6 (ATF6) – each containing a luminal domain that allows their activation by recognition of misfolded proteins [2, 3].

IRE1α contains a serine/threonine kinase and an endoribonuclease domain in its cytoplasmic domain. After activation, the latter splices the mRNA of the transcription factor XBP1, causing a shift in the reading frame and allowing for translation of the “spliced” transcription factor XBP1s, which enters the nucleus and controls the production of a large number of ER chaperones as well as proteins involved in ER-associated protein degradation (ERAD), amongst others [2, 4]. Moreover, in a process called “regulated IRE1-dependent decay of mRNA (RIDD)” IRE1α can cleave additional ER-localized mRNAs [5]. Activated PERK phosphorylates and thereby inactivates the translation initiation factor eIF2α, resulting in a global attenuation of protein synthesis. Paradoxically, phosphorylated eIF2α promotes translation of several proteins, a prominent one being the pro-apoptotic transcription factor ATF4 [2, 6]. The third sensor, ATF6, upon ER stress, de-oligomerizes and translocates from the ER to the Golgi apparatus, where it is cleaved by membrane-bound proteases in its cytoplasmic domain. Cleavage results in the release of the N-terminal domain, which subsequently enters the nucleus and acts as a transcription factor [2, 7].

The context-dependent cooperation of these sensors and the respective transcription factors allow for cellular responses ranging from adaptation to apoptosis [2]. This apoptotic switch has been utilized already for the treatment of the plasma cell malignancy called multiple myeloma (MM), the cells of which exert marked secretory capacity and constitutively depend on the UPR [8, 9]. To induce UPR-directed apoptosis, the proteasome inhibitor bortezomib (BZ), which has been established as a standard of care for MM patients, was applied to impede degradation of misfolded proteins by the proteasome in the ERAD pathway, thus enhancing the protein load of the ER and tipping the balance of the stress response towards apoptosis. In addition to proteasome inhibitors, the glycosylation inhibitor tunicamycin (TM), the inhibitor of the sarcoplasmic/endoplasmic reticulum Ca^2+^-dependent ATPase (SERCA), thapsigargin, as well as the reducing agent dithiothreitol (DTT) are frequently used to artificially induce the UPR [10].

Systemic mastocytosis (SM) is a clonal mast cell (MC) disease exhibiting various characteristics from indolent mastocytosis to aggressive mastocytosis and MC leukemia (MCL) [11, 12]. SM features abnormal proliferation and accumulation of MCs in various tissues and organs [11, 12]. The receptor tyrosine kinase KIT plays a central role in MC biology by promoting development, survival, proliferation, chemotaxis as well as proinflammatory mediator production and release [12, 13]. Mutations in KIT can be found in most cases of SM, with KIT^D816V^ being the most prevalent and detectable in more than 80 % of patients [11, 12]. Since KIT^D816V^ may be found in all variants of SM (from indolent SM to MCL) with comparable prevalence [14], mutations in additional genes might be present causing the differences in disease severity. Indeed, mutations in genes encoding for various signaling proteins, epigenetic regulators, transcription factors, and splicing factors were identified [15-17]. Moreover, a thorough molecular analysis of myeloid progenitor cells in multi-mutated advanced SM revealed KIT^D816V^ as a late event [18]. Nevertheless, it is understood that KIT^D816V^ is important in SM regarding MC proliferation and survival. Inhibition of KIT^D816V^ by established tyrosine kinase inhibitors (TKIs), however, faces several problems. KIT^D816V^ is resistant to the first generation TKI imatinib and high concentrations of the second generation TKI nilotinib are required for its inhibition [19, 20]. Two further second generation TKIs, dasatinib and midostaurin, and the third generation TKI ponatinib can suppress KIT^D816V^–driven cellular activities *in vitro* [21-24], however, the determination of drug-protein interaction profiles as well as phosphoproteome analyses revealed restricted selectivity, offering the possibility of unwanted side effects [25-28]. Nevertheless, recent studies revealed effectiveness of nilotinib and midostaurin in a number of patients with advanced systemic mastocytosis, including highly fatal MCL [29, 30]. However, further kinases except KIT, such as the SRC family kinase LYN, the TEC family kinase BTK, and the mitosis-regulating serine/threonine kinase PLK1, have been demonstrated to be involved in the regulation of proliferation and survival of MCL cell lines as well as patient cells [31, 32], which might account for patient- and situation-specific restricted efficacy of the above mentioned TKIs. Hence, further TKI-independent therapies or the use of synergistically acting drug combinations should be developed.

In this study, we have approached the importance of the UPR in MCL and analyzed the efficacy of various UPR inhibitors and pharmacological inducers of ER stress to suppress proliferation and survival of the KIT^V560G,D816V^-positive human MCL cell line HMC-1.2. In addition, we unraveled the potency of a combination of BZ and the JNK inhibitor JNK-IN-8 to efficiently induce apoptosis in KIT^D816V^-positive MCL cells.

## RESULTS

### Inhibition of the IRE1α arm of the UPR suppresses proliferation and survival of HMC1.2 cells

**Figure 1:**
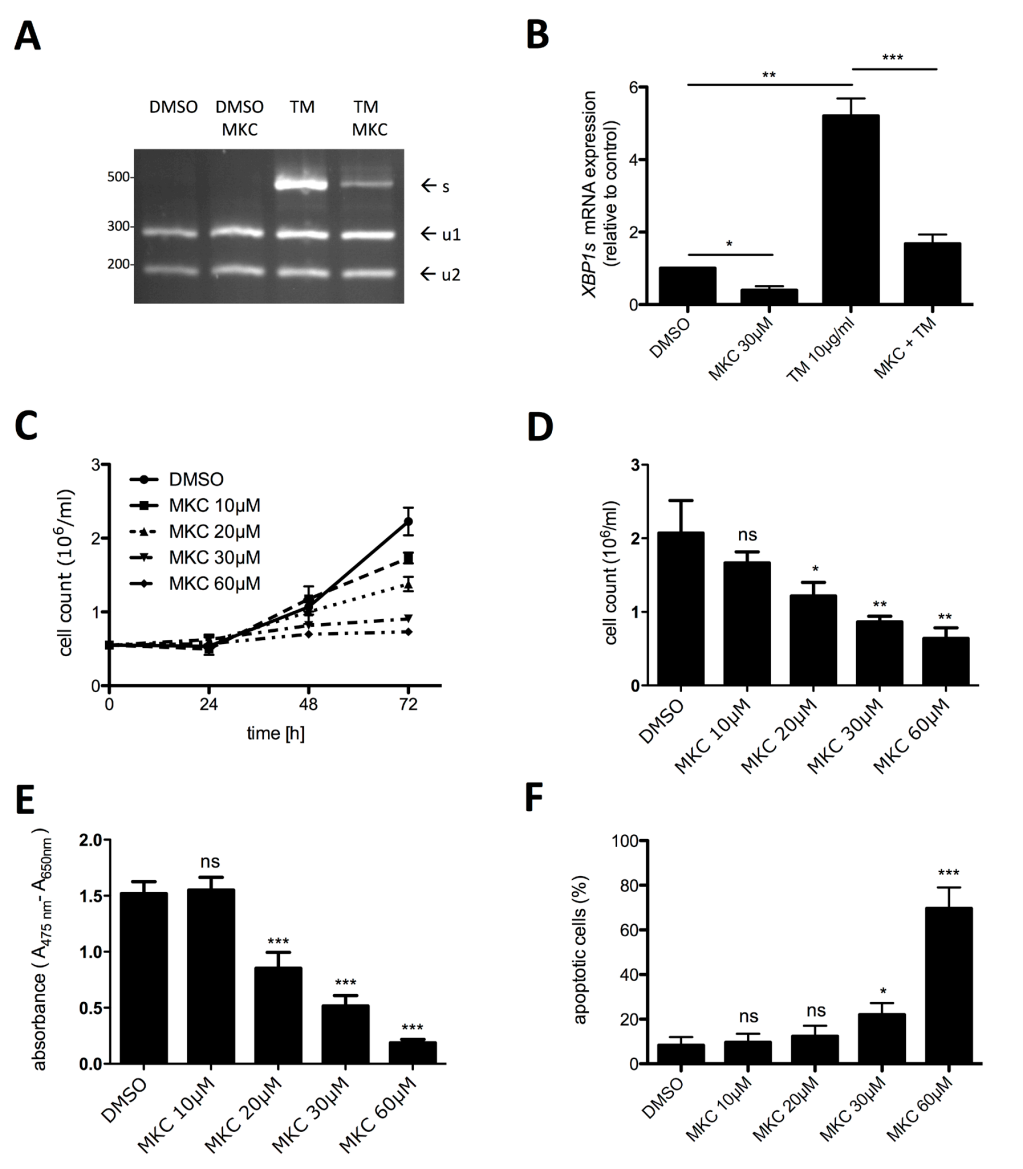
Inhibition of active IRE1α by MKC-8866 mainly suppresses proliferation of HMC-1.2 cells **A**. Expression of spliced *XBP1* mRNA was evaluated by an *XBP1* splicing detection assay. HMC-1.2 cells were pre-incubated with vehicle (DMSO) or 30µM MKC-8866 (MKC) for 1h followed by 6h treatment with10µg/ml TM. Generated cDNA was used to amplify *XBP1* by PCR. XBP1u amplicon digestion by PstI resulted in two fragments (u1, u2). Size of XBP1s amplicon lacking PstI sites was 473bp. **B**. HMC-1.2 cells were treated for 1h with vehicle (DMSO) or 30µM MKC followed by 6h treatment with10µg/ml TM. *XBP1s* expression was evaluated by RT-qPCR and normalized to *HPRT*. (n=3) **C**. Proliferation was measured by Casy cell counter every 24h for up to 72h. HMC-1.2 cells were treated with vehicle (DMSO) or with the indicated concentrations of MKC. Proliferation graphs of a representative experiment are shown. **D**. Statistical analysis of cell counts after 72h of independent experiments as measured in C. (n=3) **E**. Metabolic activity was measured by XTT assay after 72h treatment with the indicated concentrations of MKC (or vehicle). (n=5) **F**. Cell viability was measured by FACS analysis of Annexin V/ propidium iodide stained HMC-1.2 cells treated for 72h with vehicle (DMSO) or the indicated concentrations of MKC. (n=3) Data show mean ± SD from n ≥ 3 independent experiments. Student’s *t*-Test and one-sample *t*-Test were performed to calculate the p-values. *p < 0.05, **p < 0.01, ***p < 0.001.

In a situation-dependent manner, the UPR can result in an adaptive, pro-homeostatic or in a terminal, pro-apoptotic cellular response. Cells that rapidly proliferate and possess developed secretory functions are particularly dependent on a functional adaptive UPR to cope with the synthetic demand of the ER. Thus, we interrogated the KIT^V560G,D816V^-positive human MCL cell line HMC-1.2 for a constitutively active UPR by determining activation of the UPR sensor IRE1α. Occurrence of spliced *XBP1* mRNA (*XBP1s*) was taken as the read-out for IRE1α activation. First, we applied an *XBP1* splicing detection assay involving mRNA amplification by RT-PCR followed by diagnostic restriction digest. As a positive control, cells were treated with TM for 6 h. As expected, TM induced a strong splicing of *XBP1* mRNA, which was suppressed by the IRE1α inhibitor MKC-8866, which targets the endonuclease domain of IRE1α (Figure 1A). In single experiments, a faint band of *XBP1s* was already detectable in proliferating HMC-1.2 cells (data not shown), suggesting a weak basal activity of IRE1α in HMC-1.2 cells. The data obtained with the *XBP1* splicing detection assay were corroborated using *XBP1s*-specific RT-qPCR (Figure 1B). Owing to the enhanced sensitivity of the RT-qPCR approach, significant reduction of basal *XBP1s* mRNA by MKC-8866 was measurable, indicating once more the basal activation of IRE1α in proliferating HMC-1.2 cells. Noteworthy, constitutive activation of an UPR is not a feature of every cell type. Assuming that IRE1α activity is needed to promote growth of HMC-1.2 MCL cells, we next investigated if blocking IRE1α activity might confer inhibition of proliferation. HMC-1.2 cells were treated with increasing concentrations of MKC-8866 (10 – 60 µM) or vehicle control and cell numbers were determined every 24h using an analytical cell counter. Indeed, inhibition of IRE1α resulted in significant suppression of HMC-1.2 proliferation after 72h treatment (Figure 1C & 1D). To verify these data and to combine them with information on metabolic activity, XTT assays were performed. Incubation (72h) with MKC-8866 caused a dose-dependent decline in metabolic activity (Figure 1E). Compared to the sole determination of cell numbers (Figure 1D), a marked diminution of XTT positivity was evident from 30 µM to 60 µM of MKC-8866, suggesting appearance of an additional quality in the presence of 60 µM MKC-8866 (Figure 1E). Therefore, we analyzed induction of cell death in MKC-8866-treated HMC-1.2 cells by staining with AV/PI. Whereas 10 - 30 µM MKC-8866 induced only little cell death, 60 µM MKC-8866 caused up to 60% of AV/PI-positive cells (Figure 1F; Supplementary Figure 1A), paralleling the decline in XTT positivity at this concentration of the inhibitor (Figure 1E), thus corroborating qualitatively differential effects dependent on inhibitor concentration. Comparable results were obtained analyzing the effects of MKC-8866 on KIT^V560G^-positive HMC-1.1 cells (data not shown).

**Figure 2:**
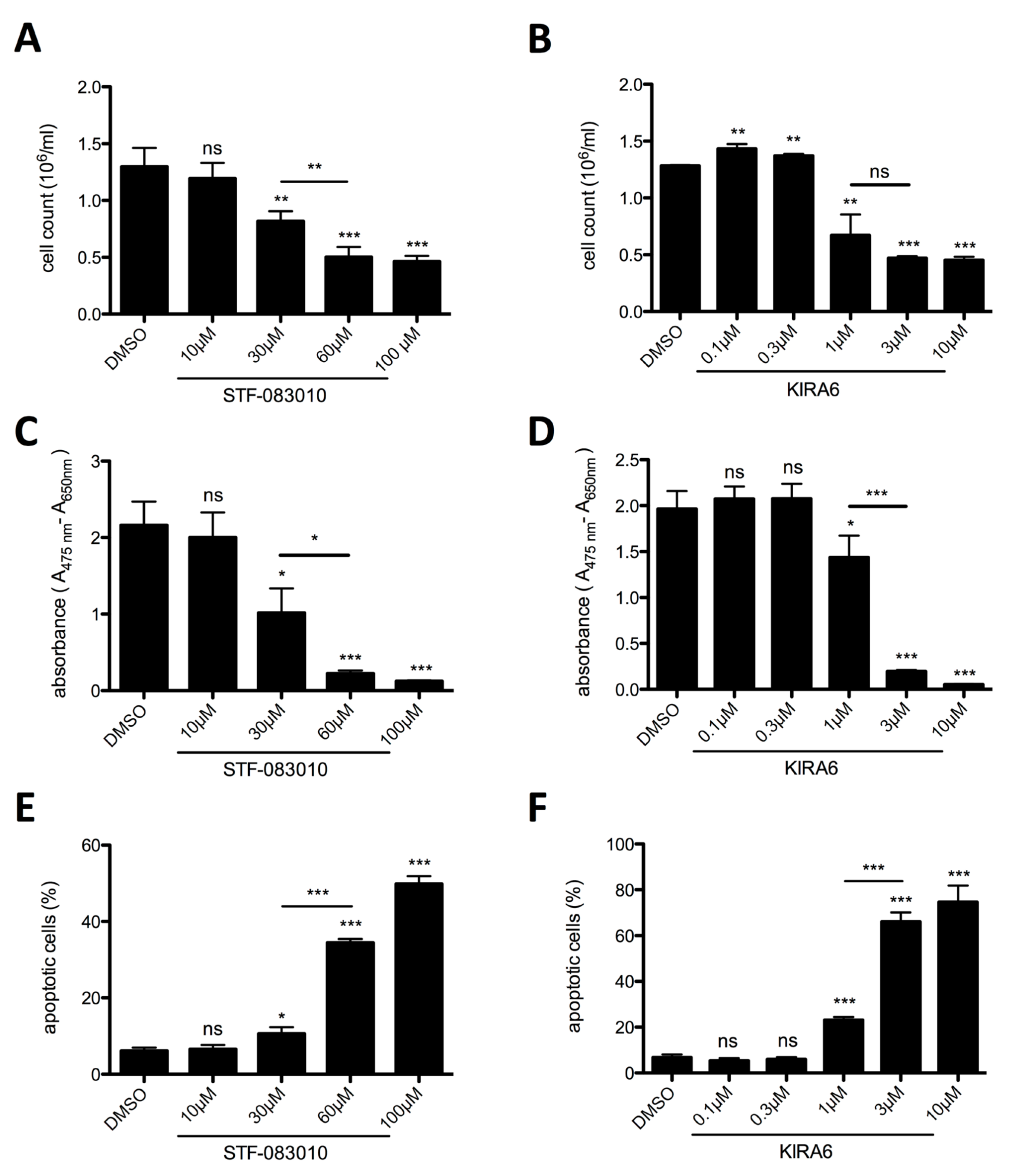
IRE1α endonuclease and kinase inhibition restrains proliferation and survival of HMC-1.2 **A**, **B**. Proliferation was measured by Casy cell counter after 72h. HMC-1.2 cells were treated for 72h with vehicle (DMSO) or with the indicated concentrations of STF-083010 (STF) (A, n=4) or KIRA6 (B, n=3). **C**, **D**. Metabolic activity after 72h treatment with indicated concentrations of STF (C, n=3) or KIRA6 (D, n=3) was measured by an XTT proliferation kit. **E**. Cell viability after 72h treatment with indicated concentrations of STF (E, n=3) or KIRA6 (F, n=3) was measured by FACS analysis of Annexin V/ propidium iodide stained HMC-1.2 cells. Data shown are mean ± SD from n ≥ 3 independent experiments. Student’s *t*-Test and one-sample *t*-Test were performed to calculate the p-values. *p < 0.05, **p < 0.01, ***p < 0.001.

To corroborate the effect of MKC-8866-mediated IRE1α inhibition, two additional pharmacological inhibitors of IRE1α were tested: the IRE1α endonuclease-specific inhibitor STF-083010 [33] and the ATP-competitive IRE1α kinase-inhibiting RNase attenuator KIRA6 [34]. Both inhibitors significantly and dose-dependently suppressed proliferation determined by cell counting (Figure 2A & 2B) and metabolic activity of HMC-1.2 cells as measured by XTT assays (Figure 2C & 2D). In line with the data obtained with MKC-8866, both STF-083010 and KIRA6 induced cell death at higher concentrations in an escalating manner (STF-083010: compare 30 and 60/100 µM; KIRA6: compare 1 and 3/10 µM) (Figure 2E & 2F; Supplementary Figure 1B & 1C). This was intriguing all the more since an effective blockade of *XBP1* splicing was already obtained using the lower concentrations of both inhibitors (STF-083010: 30 µM; KIRA6: 1 µM) (Supplementary Figure 2). Particularly KIRA6 was a potent inducer of cell death, potentially owing to its dual activity inhibiting both the endonuclease activity as well as the kinase activity of IRE1α. In conclusion, proliferation and survival of the human MCL cell line HMC-1.2 rely on a functional UPR, in particular signaling from the UPR sensor IRE1α, and indicate the potential of IRE1α inhibitors as pharmacological treatment for patients suffering from MCL.

### Constitutive activation of PERK in HMC-1.2 cells does not support proliferation and survival

**Figure 3:**
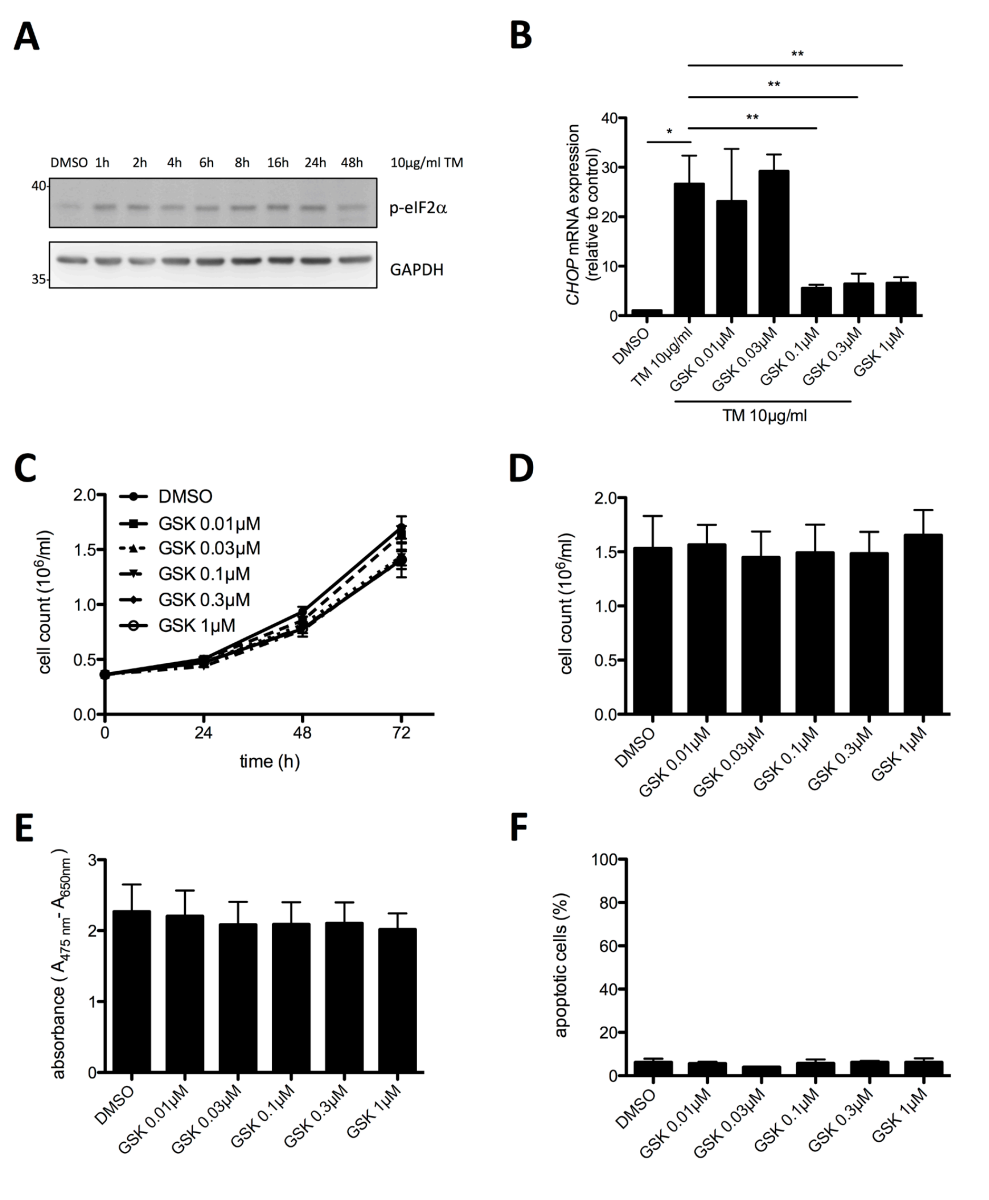
Proliferation and survival of HMC-1.2 cells is independent on PERK pathway activation **A**. HMC-1.2 cells were treated with vehicle (DMSO) or 10 µg/ml TM for the indicated time points and phosphorylation of eIF2α was detected by Western blot. GAPDH served as loading control. **B**. Inhibition of TM-induced *CHOP* expression by GSK2606414 (GSK) was evaluated by RT-qPCR. HMC-1.2 cells were pretreated for 1h with vehicle (DMSO) or the indicated concentrations of GSK followed by TM treatment for 6h. *CHOP* mRNA expression was normalized to *HPRT*. (n=3) **C**. Impact of GSK on HMC-1.2 proliferation was measured by automated Casy cell counter every 24h for up to 72h. HMC-1.2 cells were treated for 72h with vehicle (DMSO) or with the indicated concentrations of GSK. Proliferation graphs of a representative experiment are shown. **D**. Statistical analysis of cell counts after 72h of independent experiments as measured in C. (n=3) **E**. Effect of the indicated concentrations of GSK on metabolic activity after 72h was measured by an XTT proliferation kit. (n=3) **F**. Cell viability was measured by FACS analysis of Annexin V/ propidium iodide stained HMC-1.2 cells treated for 72h with vehicle (DMSO) or the indicated concentrations of GSK. (n=3) Data shown are mean ± SD from n ≥ 3 independent experiments. Student’s *t*-Test and one-sample *t*-Test were performed to calculate the p-values. *p < 0.05, **p < 0.01, ***p < 0.001.

Motivated by the finding of basal UPR activity in HMC-1.2 cells, we investigated activation of the second UPR sensor, PERK, in proliferating HMC-1.2 cells. Phosphorylation of the translation initiation factor eIF2α was taken as a read-out for PERK activity. Phospho-specific immunoblot analysis of eIF2α in lysates of proliferating, vehicle (DMSO)-treated HMC-1.2 cells showed basal phosphorylation of eIF2α suggesting constitutive activation of the UPR (Figure 3A). Enforced activation of the UPR by treatment with the glycosylation inhibitor TM resulted in enhanced eIF2α phosphorylation, indicating that the output of PERK was not maxed in proliferating HMC-1.2 cells (Figure 3A). Using RT-qPCR we found that *CHOP* mRNA was markedly enhanced after 24h in TM-treated HMC-1.2 cells, which was significantly reduced by the PERK inhibitor GSK2606414 (Figure 3B). Treatment with 0.1 µM GSK2606414 resulted in a near to maximum suppression of TM induced *CHOP* mRNA production (Figure 3B) as well as corresponding eIF2α phosphorylation (Supplementary Figure 3). Having identified the effective concentrations of GSK2606414, we next investigated if blocking PERK activity might inhibit proliferation of HMC-1.2 cells. Hence, cells were treated with increasing concentrations of GSK2606414 (0.01 – 1 µM) or vehicle and cell numbers were determined every 24h using an analytical cell counter. Inhibition of PERK had no significant effect on proliferation of HMC-1.2 cells after 72h treatment (Figure 3C & 3D). This was corroborated by XTT assays measuring metabolic activity (Figure 3E) as well as by analysis of apoptosis/cell death in GSK2606414-treated cells by staining with annexin V and propidium iodide (AV/PI) (Figure 3F). In conclusion, the PERK pathway of the UPR appears slightly active in proliferating HMC-1.2 cells, however, an involvement in proliferation and survival, in comparison to the IRE1α/XBP1s pathway, was not observed.

### Proteasome inhibition suppresses proliferation and survival of human MCL cells

**Figure 4:**
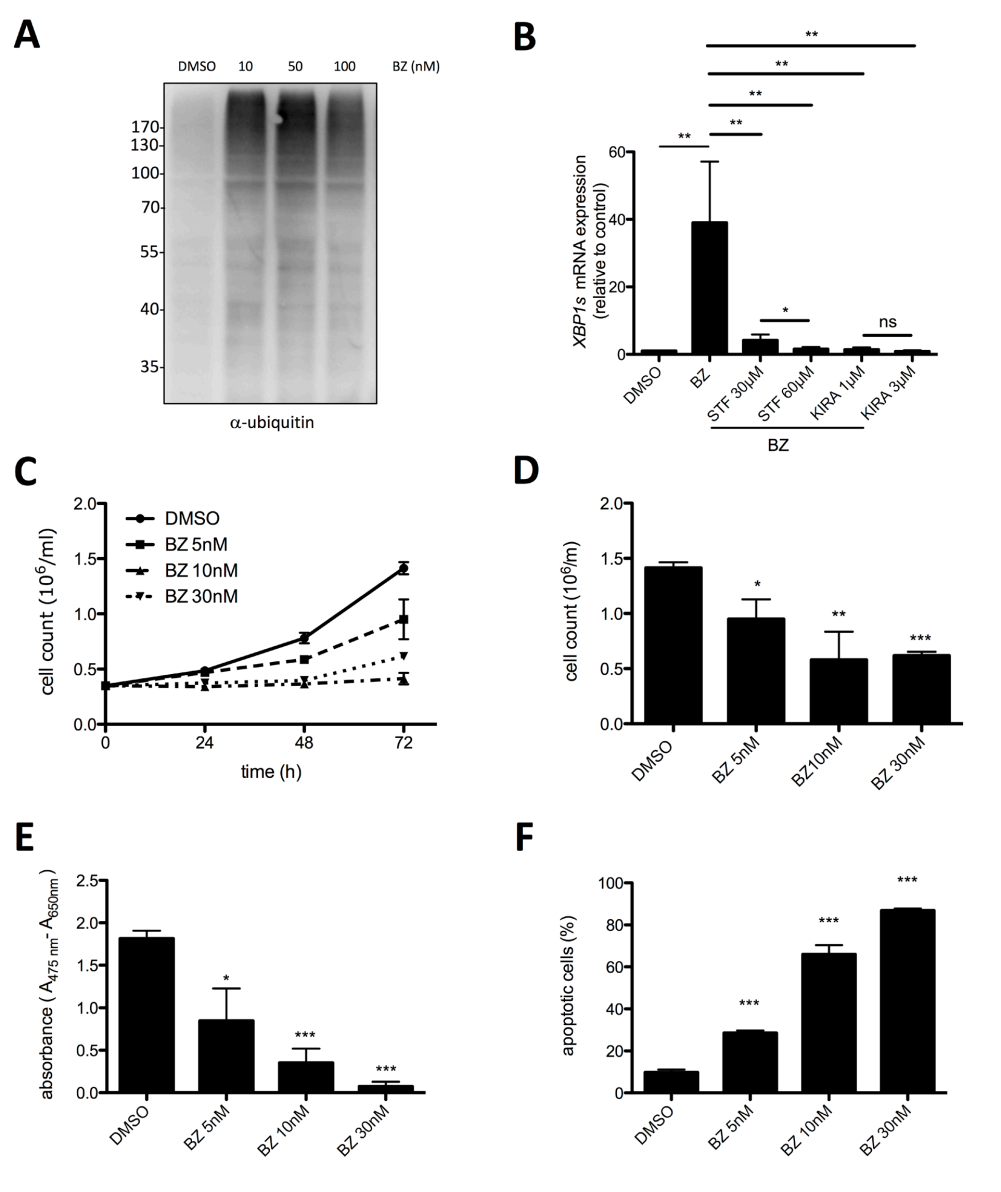
Proteasome inhibitor bortezomib suppresses proliferation and survival of HMC-1.2 cells **A**. Bortezomib (BZ) induced pan-ubiquitination of proteins (shown as smear) in HMC1.2 cells after 24h treatment with the indicated concentrations. Ubiquitination was detected by Western blotting. **B**. HMC-1.2 cells were treated with vehicle (DMSO) or STF or KIRA6 for 60min followed by a 30 nM BZ treatment for 24h. *XBP1s* mRNA expression was measured by RT-qPCR and normalized to *HPRT*. (n=5) **C**. Proliferation was measured by Casy cell counter every 24h for up to 72h. HMC-1.2 cells were treated with vehicle (DMSO) or with the indicated concentrations of BZ. Proliferation graphs of a representative experiment is shown. **D**. Statistical analysis of cell counts after 72h of independent experiments as measured in C. (n=3) **E**. Metabolic activity was measured by XTT assay after 72h treatment with the indicated concentrations of BZ (or vehicle). **F**. Cell viability was measured by FACS analysis of Annexin V/ propidium iodide stained HMC-1.2 cells treated for 72h with vehicle (DMSO) or the indicated concentrations of BZ. (n=3) Data shown are mean ± SD from n ≥ 3 independent experiments. Student’s *t*-Test and one-sample *t*-Test were performed to calculate the p-values. *p < 0.05, **p < 0.01, ***p < 0.001.

Our data so far demonstrated that survival of HMC-1.2 MCL cells is supported by the constitutive activation of the UPR, especially the IRE1α arm. An important mechanism of cells to cope with rising levels of unfolded proteins is ERAD that depends on a functional ubiquitin proteasome system. Proteasome inhibitors, particularly the FDA-approved BZ (Velcade, originally codenamed PS-341), are used in MM cells and patients to block the ERAD resulting in enhanced accumulation of (misfolded) proteins in the ER, and thus to induce terminal UPR [35, 36]. On the other side, various cancer cell lines have been shown to be resistant to BZ [37]. Thus, the possibility to reduce HMC-1.2 proliferation and survival by BZ treatment was thoroughly investigated. Measurement of total protein ubiquitination by immunoblotting of whole-cell lysates with ubiquitin-specific antibodies as readout for proteasome inhibition, proved 10 nM BZ to be sufficient for an optimal response (Figure 4A). Moreover, 10 nM BZ triggered significant activation of the IRE1α path of the UPR as measured by RT-qPCR of *XBP1s* (Figure 4B). Accordingly, BZ-induced *XBP1s* generation was suppressed by STF-083010 and KIRA6 (Figure 4B). Next, proliferation of HMC-1.2 cells in the absence or presence of BZ was analyzed. Treatment with 5-30 nM BZ for 24-72h was found to completely block proliferation (Figure 4C & 4D). This was in agreement with the suppression of metabolic activity measured by XTT assay, which suggested induction of cell death by BZ treatment (Figure 4E). Hence, by determining AV/PI double-positivity, BZ-triggered cell death was analyzed. Indeed, BZ was a potent inducer of HMC-1.2 cell death with 10 and 30 nM causing approximately 40% and 80% double-positive cells, respectively (Figure 4F; Supplementary Figure 1D). Thus, our data suggest that comparable to MM [35], BZ-mediated proteasome inhibition might be usable as a therapeutic option in KIT^D816V^-positive MCL.

### Inhibition of BZ-induced JNK activity synergistically promotes cell death of HMC-1.2 MCL cells

**Figure 5:**
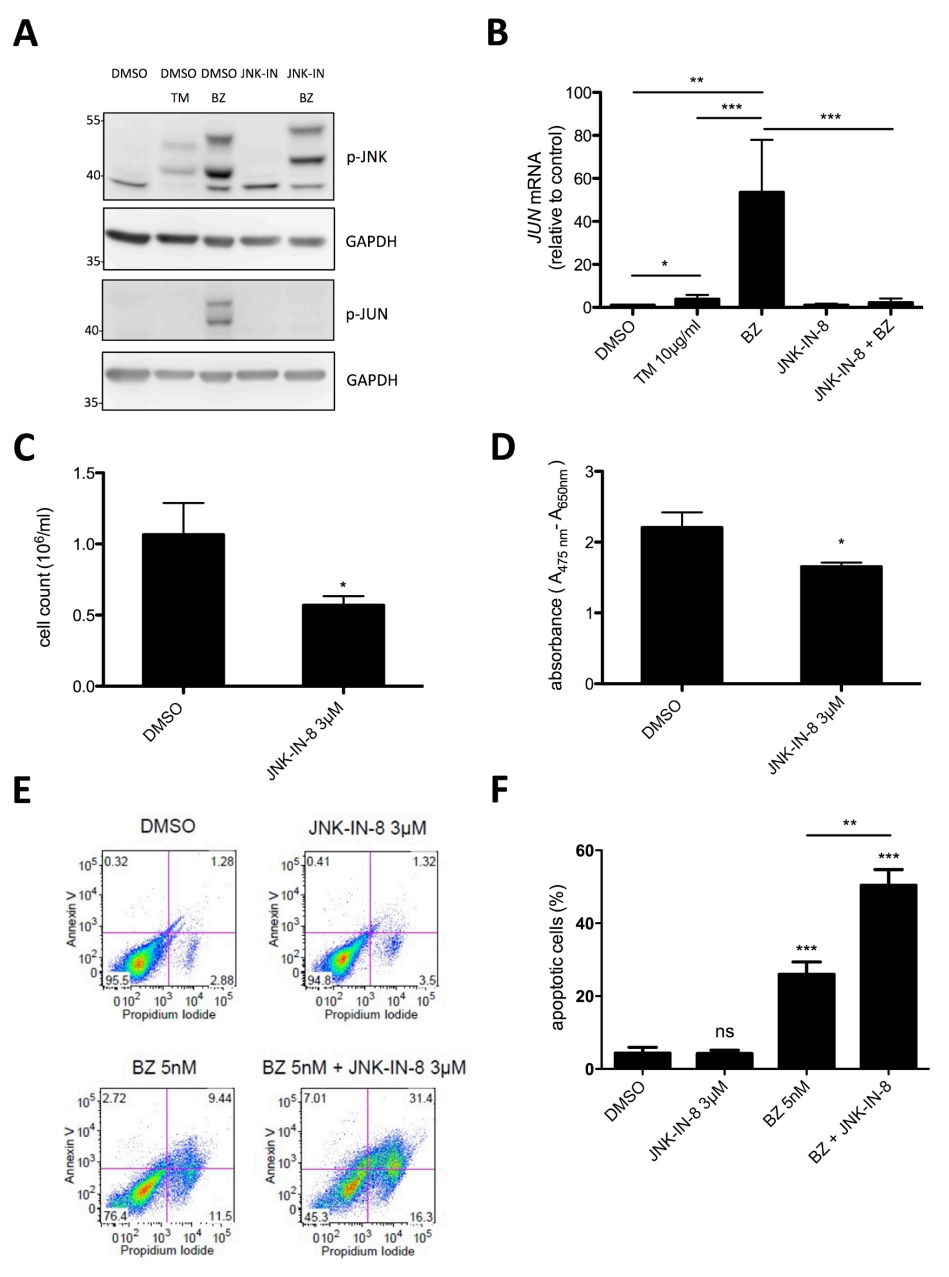
Synergistic induction of cell death by a combination of low dose BZ with JNK-IN-8 **A**. HMC-1.2 cells were pre-incubated with vehicle (DMSO) or JNK-IN-8 for 1h followed by TM (10µg/ml) or BZ (10 nM) treatment for 24h. Phosphorylation of JNK and JUN was detected by Western blotting. GAPDH served as loading control. (n=3) **B**. HMC-1.2 cells were pretreated for 1h with vehicle (DMSO) or JNK-IN-8 (3µM) followed by a 24h BZ treatment. *JUN* mRNA expression was evaluated by RT-qPCR and normalized to *HPRT*. (n=4) **C**, **D**. HMC-1.2 cells were treated with DMSO or 3µM JNK-IN-8 for 24h and proliferation (Casy cell counting) C (n=4) and metabolic activity (XTT assay) D (n=3) were measured. **E**. Cell viability was determined by FACS analysis of Annexin V/ propidium iodide stained HMC-1.2 cells treated for 72h with the indicated substances. One representative experiment is shown. **F**. Statistical analysis of the amount of apoptotic cells after 72h of independent experiments as measured in E. (n=3) Data shown are mean ± SD from n ≥ 3 independent experiments. Student’s *t*-Test and one-sample *t*-Test were performed to calculate the *p*-values. *p < 0.05, **p < 0.01, ***p < 0.001.

The stress kinase JNK has been shown to be activated in response to proteasome inhibition and to initiate an apoptotic program [38]. On the other hand, JNK has been found in MCs to promote proliferation [39, 40]. Moreover, JNK has been demonstrated in cancer cells to be able to mediate both pro-survival and pro-apoptotic signaling [41]. Providing that BZ treatment of HMC-1.2 cells results in activation of JNK, which might exert a proliferation- and/or survival-promoting role, combined use of proteasome and JNK inhibitors might be used as effective treatment of MCL. To address this question, HMC-1.2 cells were treated with 10 nM BZ for 24h and phosphorylation of JNK was measured by phospho-specific immunoblotting. Indeed, BZ induced pronounced phosphorylation of JNK, which was manifested in JNK activation since the characteristic JNK target, JUN, was phosphorylated as well (Figure 5A). Inhibition of JNK by the selective pharmacological JNK inhibitor, JNK-IN-8 [42], did not block BZ-induced JNK phosphorylation, but completely suppressed phosphorylation of JUN (Figure 5A). The markedly retarded electromobility of p-JNK from BZ- vs. BZ/JNK-IN-8-treated cells can be explained by covalent binding of JNK-IN-8 to a conserved Cys residue within JNK [42]. Moreover, BZ treatment caused marked induction of *JUN* mRNA, which was inhibited by JNK-IN-8 (Figure 5B). The observed equivalence between JNK phosphorylation and activation was not self-evident, because TM treatment of HMC-1.2 cells also induced JNK phosphorylation, however, did not cause phosphorylation of JUN (Figure 5A). This difference coincided with an altered running behavior of p-JNK in TM- vs. BZ-treated HMC-1.2 cells. Next, we analyzed the effect of treatment with JNK-IN-8 on proliferation, metabolic activity, and survival of HMC-1.2 cells. For these experiments, we used 3 µM of JNK-IN-8, a concentration that effectively suppressed JUN phosphorylation and *JUN* mRNA production in response to BZ (Figure 5A & 5B). Within 72h, JNK-IN-8 reduced proliferation of HMC-1.2 cells by approximately 40 % (Figure 5C), exerted only a minor effect on metabolic activity (Figure 5D), and did not impact on survival (Figure 5E & 5F). Intriguingly and confirming our initial assumption that BZ-induced activation of JNK is counteracting induction of cell death of HMC-1.2 cells, the combination of 3 µM JNK-IN-8 with a very low concentration of BZ (5 nM) resulted in a synergistic increase of cell death compared to BZ alone (Figure 5E & 5F). In conclusion, enhancing the cytotoxic effect of BZ-induced proteasome inhibition with concomitant JNK suppression by JNK-IN-8 might have the potential as a novel effective therapy for patients suffering from MCL.

### IRE1α mediates BZ-induced *JUN* and *CHOP* transcription

**Figure 6:**
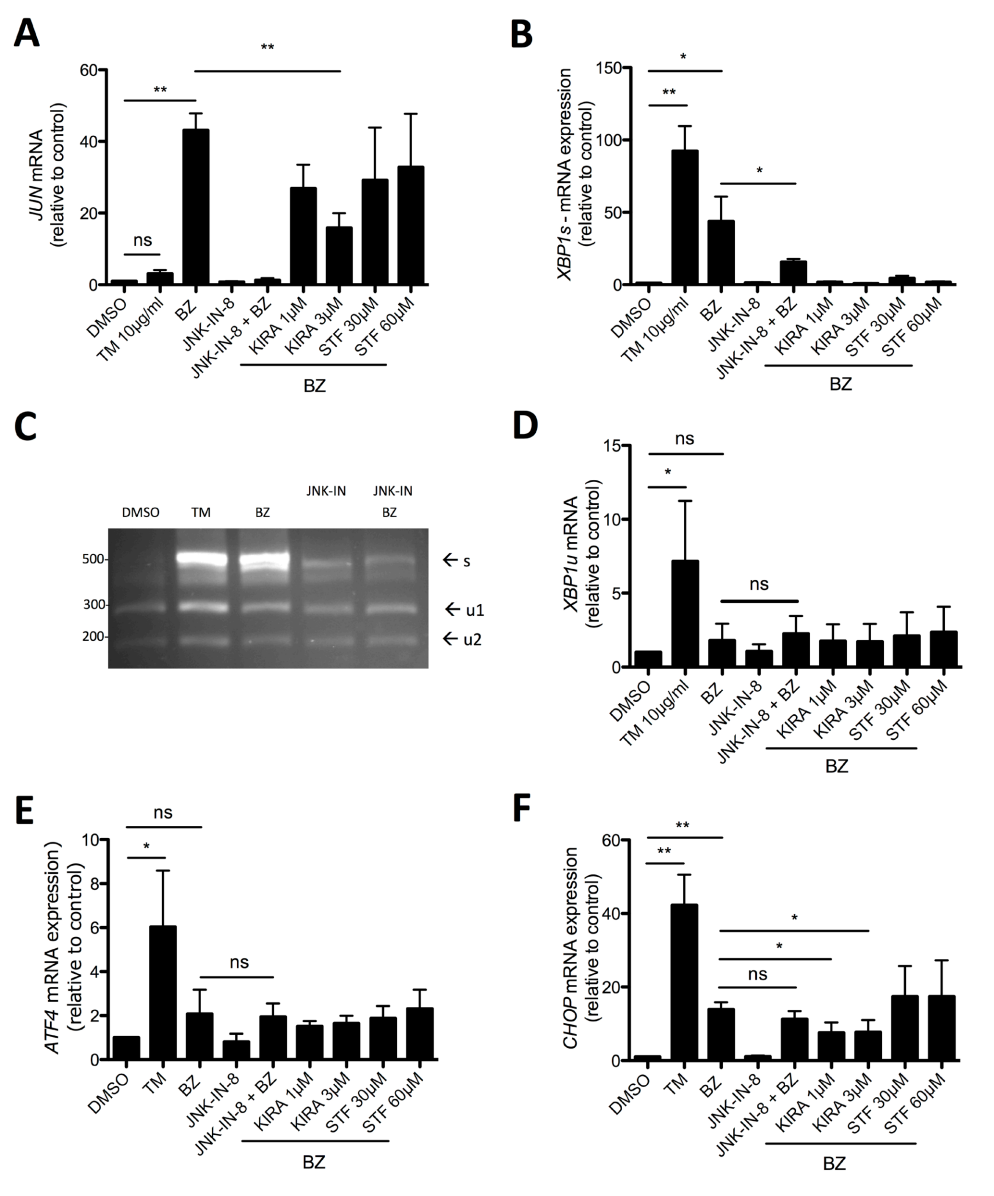
JNK signaling is involved in *XBP1* splicing **A, B, D, E, F**. HMC-1.2 cells were pretreated for 1h with vehicle (DMSO) or indicated concentrations of the inhibitors JNK-IN-8, KIRA6 or STF followed by a 30 nM BZ treatment for 24h. *JUN* A, *XBP1s* B, *XBP1u* D, *ATF4* E, and *CHOP* F mRNA expression was evaluated by RT-qPCR and normalized to HPRT. (n=4) **C**. Expression of spliced *XBP1* mRNA was evaluated by spliced *XBP1* assay as in Figure 2A. Data shown are mean ± SD from n ≥ 3 independent experiments. Student’s *t*-Test and one-sample *t*-Test were performed to calculate the *p*-values. *p < 0.05, **p < 0.01, ***p < 0.001.

Via a complex with the adaptor protein TRAF2, IRE1α was demonstrated to activate JNK [43]. Moreover, in acute myeloid leukemia JUN was recently found to be involved in the transcription of the UPR transcription factors XBP1 and ATF4 [44]. As shown before, in comparison to TM, BZ induced strong production of *JUN* mRNA and pretreatment with the selective JNK inhibitor JNK-IN-8 suppressed BZ-induced *JUN* transcription (Figure 6A). Interestingly, the IRE1α kinase/endonuclease inhibitor KIRA6 significantly reduced *JUN* production, whereas STF-083010, being an endonuclease inhibitor only, was unable to attenuate *JUN* transcription (Figure 6A). This suggested that IRE1α, via its kinase activity, is directly involved in the activation of the JNK/JUN pathway. Next, we analyzed if JNK is involved in the transcription as well as splicing of *XBP1*. BZ treatment of HMC-1.2 cells caused the production of *XBP1s* mRNA, which was significantly reduced by pre-incubation with JNK-IN-8 (Figure 6B). These RT-qPCR data were corroborated employing the *XBP1* mRNA splicing assay (Figure 6C). This assay, in addition to *XBP1s*, allows the evaluation of changes in *XBP1u*. However, no obvious differences in the amounts of *XBP1u* were observed between BZ-treated cells pre-incubated with vehicle (DMSO) or JNK-IN-8 (Figure 6C), indicating involvement of JNK in splicing rather than production of *XBP1* in BZ-treated HMC-1.2 cells. This was verified measuring the amount of *XBP1u* mRNA using RT-qPCR (Figure 6D). Finally, the role of IRE1α and JNK with respect to BZ-induced *ATF4* and *CHOP* transcription was investigated. Interestingly, compared to TM treatment, BZ did not cause a significant induction of *ATF4* mRNA; this was not influenced by pharmacological blockade of JNK or IRE1α (Figure 6E). This is in agreement with data by Nawrocki et al., who demonstrated PERK inhibition by BZ treatment in pancreatic cancer cells [37]. Unexpectedly and in contrast to *ATF4*, BZ did induce transcription of *CHOP*, though less pronounced than TM. This induction was weakly, but significantly attenuated by KIRA6, however not by STF-083010 and JNK-IN-8, suggesting a JNK-independent function of the activated IRE1α kinase domain (Figure 6F). So far, we cannot exclude participation of ATF6 in BZ-triggered *CHOP* transcription [45, 46]. In conclusion, JNK is mainly involved in BZ-induced *XBP1s* production and hence promotion of proliferation and survival, causing enhanced vulnerability for combined BZ and JNK-IN-8 treatment.

## DISCUSSION

For cancer therapy, both blockade of adaptive UPR as well as exaggeration of ER stress resulting in terminal UPR might be employed. Our data concentrating on MCL and utilizing the KIT^V560G,D816V^-positive MCL cell line HMC-1.2 as a model showed for the first time the efficacy of both approaches with respect to induction of cytostatic and/or cytotoxic responses. Inhibition of the IRE1α arm of the UPR by different types of inhibitors (MKC-8866, STF-083010, and KIRA6) suppressed proliferation at concentrations needed for blockade of IRE1α-mediated *XBP1* splicing. At higher concentrations, these inhibitors triggered apoptosis by terminal UPR. Enhanced activation of the UPR by proteasome inhibition, which blocks the ERAD pathway and promotes the excessive accumulation of proteins in the ER, caused a marked cytotoxic response. Additional inhibition of the compensating activation of the kinase JNK allowed for synergistic induction of cell death at comparatively low inhibitor concentrations. Hence, our data suggest that the UPR can be addressed as an attractive and effective therapeutic target against KIT^D816V^-positive MCL. Comparable low (2-10 nM), pharmacologically achievable concentrations of BZ were already shown to suppress growth of MM cells as well as to sensitize them to conventional chemotherapeutic agents [35, 47].

Cells of the plasma cell neoplasm, MM, are dependent on a functioning IRE1α/XBP1 arm of the UPR. Inhibition of the RNase activity of IRE1α by the small-molecule inhibitor STF-083010 was demonstrated to have cytotoxic activity against human MM cells in a xenograft model as well as ex vivo [33]. Moreover, the covalent IRE1α inhibitor 4µ8C attenuated growth of MM cell lines, however, was not associated with acute toxicity [48]. Enhancing ER stress by blocking proteasome activity using BZ (PS-341) was shown to be cytostatic and cytotoxic to human MM cell lines as well as freshly isolated patient MM cells [35]. BZ treatment lead to *XBP1* splicing and the IRE1α inhibitor MKC-3946, which, by its sole use, modestly inhibited growth of MM cells, could significantly enhance BZ-induced cell death [49]. Motivated by these reports, we analyzed the importance of the UPR for growth and survival of human MCL cells using the KIT^V560G,D816V^-positive MCL cell line HMC-1.2. Employing three different IRE1α inhibitors, cytostatic as well as cytotoxic activities could be observed dependent on the inhibitor concentrations applied. These inhibitors were MKC-8866 and STF-083010 blocking the RNase activity of IRE1α [33] as well as KIRA6, an ATP-competitive IRE1α kinase inhibitor allosterically co-inhibiting IRE1α´s RNase activity by breaking respective oligomers [34].

Inhibitor concentrations that maximally blocked *XBP1* splicing caused strong suppression of HMC-1.2 proliferation, however, only weak cytotoxic effects were observable. On the other hand, concentrations higher than needed for the full blockade of *XBP1* splicing were able to induce significant apoptosis. The reason for this qualitative difference, the switch from a cytostatic to a cytotoxic response, has not been clarified yet. One possibility could be the potential need for higher concentrations of IRE1α inhibitors for the suppression of RIDD. While this cannot be excluded for the two IRE1α RNase inhibitors, MKC-8866 and STF-083010, it appears rather unlikely for KIRA6 in the light of work from Papa and coworkers [34]. In their experiments, the IC50 of KIRA6 for RIDD inhibition was lower than the IC50 for inhibition of *XBP1* splicing. Another possibility might be cytotoxic side effects of these inhibitors at higher concentrations. KIRA6 as an ATP-competitive inhibitor might additionally block other kinases possibly involved in pro-survival signaling. Indeed, our data indicate that KIRA6, either directly or indirectly, is able to suppress phosphorylation/activation of JNK. Moreover, all three inhibitors, at higher concentrations, might interact with completely different targets causing pro-apoptotic signals. The principle variability of inhibitors has previously been shown convincingly by Rix et al, who found by chemical proteomic profiling that the oxidoreductase NQO2 is inhibited at physiologically relevant concentrations by the otherwise rather selective tyrosine kinase inhibitors, imatinib and nilotinib [27]. A further example of IRE1α-independent KIRA6 activity might underlie the small, though significant pro-proliferative effect of low concentrations of this IRE1α inhibitor (Figure 2B). At this point, the involved target of KIRA6 is not known. Ghosh et al. tested a panel of seven Ser-/Thr-kinases for potential cross-reactivity of KIRA6 [34]. Among these kinases only IRE1α was inhibited in *in vitro* kinase assays. However, the human kinome is considerably bigger and it cannot be excluded that one or more of the many non-tested kinases i) can be inhibited by KIRA6 and ii) has anti-proliferative effects. At higher concentrations of KIRA6, the measured pro-proliferative effect would then be outcompeted by the pro-apoptotic effect of complete IRE1α inhibition and suppression of XBP1s expression.

After demonstrating the suitability of inhibition of an adaptive UPR with respect to suppression of MCL cell proliferation and induction of cell death, we next sought to address the possibility of switching an adaptive to a terminal UPR. To reach this, we applied the 26S proteasome inhibitor BZ [50], and found marked triggering of an apoptotic response at reasonable low inhibitor concentrations, which were also found active in the treatment of MM cells [35]. Interestingly, proteasome inhibition has been demonstrated to induce phosphorylation/activation of the MAPK JNK [51, 52], which promoted the cytotoxic response ignited by blockade of the proteasome. In MCs, however, JNK has been found to promote proliferation [39, 40]. In addition, pro-survival SOX4-dependent upregulation of JNK1 (*MAPK8*) was demonstrated as a critical factor in acute lymphoblastic leukemia [53]. We hypothesized that BZ-induced JNK activation might be compensatory for the MCL cells and additional inhibition of JNK could thus result in enhanced, synergistic cell death of MCL cells. Indeed, combined use of BZ and the selective JNK inhibitor JNK-IN-8 was most effective in causing cell death of HMC-1.2 cells, indicating that in the case of MCL, proteasome inhibition causes pro-proliferative/anti-apoptotic JNK activation. Since active JNK was able to induce *JUN* transcription as well as JUN production in HMC-1.2 cells, a pro-survival role of JUN might be envisaged. Interesting in this respect is a recent publication by Zhou et al., who showed a pro-survival role of JUN in acute myeloid leukemia, multiple subtypes of which frequently overexpress JUN [44]. JUN was demonstrated to bind to promoters of several UPR effectors, such as XBP1 and ATF4. However, in HMC-1.2 cells *ATF4* upregulation upon BZ treatment was not observed; moreover, inhibition of JNK did not affect expression of *XBP1u*, suggesting cell type-specific regulatory mechanisms in the UPR.

**Figure 7:**
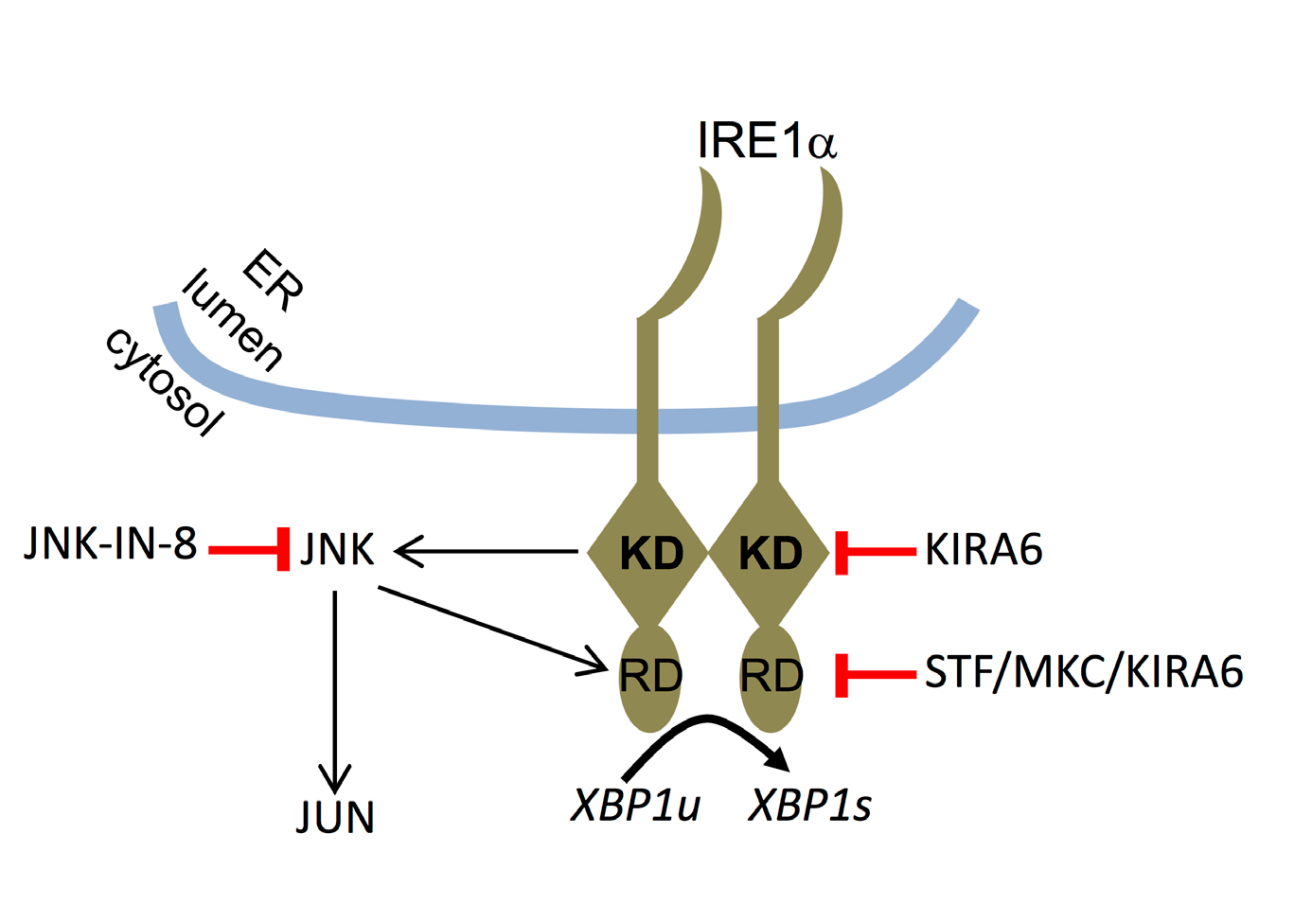
Model of functional interaction between IRE1α and JNK Upon recognition of unfolded proteins in the ER lumen, IRE1α is activated via oligomerization. Its kinase domain (KD) contributes to the activation of its RNase domain (RD) resulting in splicing of *XBP1u* to yield *XBP1s*. Moreover, the IRE1α KD mediates activation of the JNK pathway with JNK, in turn, controlling the activity of the IRE1α RD. The targets of the pharmacological inhibitors used in this study are indicated.

Interestingly, our data in HMC-1.2 cells hint at a mutual functional dependence of IRE1α and JNK. While the kinase activity of IRE1α promotes activation of JNK, JNK appears to be able to facilitate the endonuclease activity of IRE1α (Figure 7). JNK has been reported to specifically phosphorylate serine and threonine residues, which are followed by a proline residue. In several JNK targets, such as in JUN and JUND, proline is followed by an acidic amino acid [54]. Intriguingly and in line with the proposed model (Figure 7), T973 in the endonuclease domain of IRE1α has been reported to be phosphorylated [55] and resides in the context of the sequence T–P–D and thus would fulfill both location in the endonuclease domain and presence of a JNK target-specific sequence. It will be important to explore the phosphorylation of T973 by JNK and further analyze the IRE1α (kinase) - JNK - IRE1α (endonuclease) circuit in further leukemia cells.

Similar to the IRE1α arm of the UPR, the PERK arm, depending on the strength of ER stress, is able to induce both an adaptive and a terminal response. The respective adaptive response involves phosphorylation of eIF2α preventing the formation of the ternary initiation complex consisting of unphosphorylated eIF2α, GTP, and tRNA^Met^, thus inhibiting general protein synthesis. The terminal response involves selective translation of the transcription factor ATF4 resulting, amongst others, in the induction of the proapoptotic transcription factor CHOP. ATF4 has also been shown recently to induce autophagy [56]. In case of increased production of mutated or misfolded proteins in the ER, as it may occur in neurodegenerative diseases, blockade of translation by eIF2α phosphorylation appears appropriate; however, such response would not be advantageous for proliferating leukemia cells. Although we detected basal phosphorylation of eIF2α in HMC-1.2 cells, pharmacological inhibition of PERK by GSK2606414 did neither have cytostatic nor cytotoxic effects. Speaking against basal PERK activation, GSK2606414 suppressed increased eIF2α phosphorylation in response to TM treatment, however, it did not reduce basal phosphorylation of eIF2α, suggesting involvement of other kinases, like PKR or mGCN2. Moreover, as with every measurable phosphorylation event, which is the net result of kinase and phosphatase activities, eIF2α-specific phosphatases might be active in MCL cells to allow for protein production as well as to prevent ATF4/CHOP-mediated apoptosis. Further analysis of these phosphatases and their regulation in HMC-1.2 cells might enable the identification of novel drug targets in MCL.

In conclusion, we have demonstrated the importance of the UPR for the proliferation and survival of MCL cells, in particular the IRE1α-dependent arm of the UPR. We have shown that both pharmacological inhibition of IRE1α catalytic activities and exaggeration of the UPR by pharmacological stressors suppress proliferation and survival of MCL cells. Moreover, prosurvival JNK activity induced by BZ-triggered IRE1α activation is introduced as an additional drug target to boost the efficacy of BZ-induced MCL cell death. In conclusion, our data suggest that the UPR can be addressed as an effective therapeutic target against so far unsatisfactorily treatable KIT^D816V^-positive MCL.

## MATERIALS AND METHODS

### Cell culture

HMC-1.2 (KIT^V560G,D816V^) cells were kindly provided by Dr. J. Butterfield (Mayo Clinic, Rochester, MN) [57]. They were maintained in RPMI 1640 medium (Gibco, Thermo Fisher Scientific) supplemented with 10% fetal bovine serum (FBS), Hepes 1M, pH 7.0-7.6, and 10.000 units penicillin + 10 mg/ml streptomycin (all from Sigma-Aldrich) in an atmosphere containing 5% CO_2_. The medium was renewed twice a week.

### Reagents

Tunicamycin was purchased from Applichem, Bortezomib from Selleckchem, STF-083010 from Axon Medchem, KIRA6 from Cayman, and GSK2606414 as well as JNK-IN-8 from Calbiochem. DMSO was obtained from Carl Roth GmbH & Co. MKC-8866 was provided by MannKind Corporation, Valencia, CA USA.

### Western blotting and antibodies

Pelleted cells were solubilized with 0.5% NP-40 and 0.5% sodium deoxycholate in 4 °C phosphorylation solubilization buffer [58]. After normalizing for protein content, lysates were supplemented with Lämmli buffer, boiled for 5 minutes at 95°C and subjected to SDS-PAGE and subsequent Western blot analysis [59]. The following antibodies α-ubiquitin, α-p-eIF2α (Ser51), α-p-JNK (Thr183/Tyr185), and α-p-JUN (Ser63) were purchased from Cell Signaling Technology, and α-GAPDH (sc32232) from Santa Cruz.

### RNA preparation and quantitative RT-PCR

RNA from 3 x 10^6^ HMC-1.2 cells was extracted using RNeasy Plus Mini Kit (Qiagen) according to the manufacturer’s instructions. Total RNA (1 μg) was reverse transcribed using Random hexamers (Roche) and Omniscript Kit (Qiagen) according to the manufacturer’s instructions. qPCR was performed on a Rotorgene (Corbett Life Science, now Qiagen) by using SYBR green reaction mix (Bioline #QT650-02). Expression was normalized to the housekeeper HPRT. The relative expression ratio including primer efficiencies was calculated by the Pfaffl method [60]. Primer sequences and efficiency data were as follows: XBP1s fwd AAC CAG GAG TTA AGA CAG CGC TT, rev CTG CAC CTG CTG CGG ACT, 2.00; CHOP fwd GGA GCA TCA GTC CCC CAC TT, rev TGT GGG ATT GAG GGT CAC ATC, 1.98; JUN fwd TAA TCC AGT CCA GCA ACG GG, rev GTG TTC TGG CTG TGC AGT TC, 2.48; HPRT fwd TGA CAC TGG CAA AAC AAT GCA, rev GGT CCT TTT CAC CAG CAA GCT, 2.03, XBP1u fwd CAG CAC TCA GAC TAC GTG CA, rev ATC CAT GGG GAG ATG TTC TGG, 2.02.

### Apoptosis assay

Cells were seeded at a density of 3.5 x 10^5^ cells/ml and treated with the indicated substances for 72h. After treatment cells were incubated with Annexin V-Alexa Fluor 647 (Alexis Biochemicals) in culture medium for 20 min at RT in the dark. Immediately before analysis by flow cytometry, propidium iodide (1 μg/ml was added and analyzed on a FACScan (BD Biosciences).

### Proliferation assays

Cells were seeded at a density of 3.5 x 10^5^ cells/ml or 5.5 x 10^5^ cells/ml (Figure 1C/1D) and treated with different concentrations of the test substances; solvent (DMSO)-treated cells served as controls. After a 24h-treatment, cells were resuspended completely and 50 μl from each well was diluted in 10 ml PBS for automated multi-parameter cell counting using a Casy cell counter (Innovatis). Metabolic activity was measured using the XTT Cell Proliferation Kit II (XTT)(Roche). Cells were seeded in microplates at a density of 3.5 x 10^5^ cells/ml (suspension culture grade, 96 wells, flat bottom) in a final volume of 100 µl culture medium per well in a humidified atmosphere (37°C, 5% CO_2_) for 72h. After the incubation period, 50 µl of the XTT labeling mixture was added to each well (final XTT concentration 0.3 mg/ml). Incubation of the microplate was for 3 - 4 h in a humidified atmosphere (e.g., 37°C, 5% CO_2_).

Spectrophotometrical absorbance of the samples were measured using a microplate reader. The wavelength used to measure absorbance of the formazan product of the XTT assay was 475nm and the reference wavelength was 650nm. Sample values at 475nm were subtracted with medium controls (blanked) resulting in delta blanked values. Total absorbance was calculated by subtraction of delta blanked values (475nm) with their reference values at 650nm. These absorbance values (A_475nm_-A_650nm_) are shown in the respective figures.

### XBP1 mRNA splicing

RNA was isolated and reverse transcribed as above to obtain total cDNA. Then, XBP1 primers were used to amplify an XBP1 amplicon spanning the 26 nt intron from the cDNA samples in a regular 3-step PCR. Thermal cycles were: 5 min at 95 °C, 30 cycles of 30 s at 95 °C, 30 s at 60 °C, and 1 min at 72 °C, followed by 72 °C for 15 min, and a 4 °C hold. PCR was performed using h-XBP1.3 fwd: AAA CAG AGT AGC AGC TCA GAC TGC and h-XBP1.12 rev: TCC TTC TGG GTA GAC CTC TGG GAG primers. PCR fragments were then digested by PstI and separated on Ethidium Bromide containing 2% agarose gels. The restriction digest of unspliced XBP1 (XBP1u) resulted in two fragments of 290 and 183bp. The size of the XBP1s amplicon lacking PstI sites was 473bp.

### Statistical analysis

Data were generated from independent experiments. P-values were calculated by the unpaired two-tailed Student’s *t*-test or the one-sample *t*-Test using GraphPad Prism. Figures represent means ± SD of n ≥ 3 independent experiments (with n indicated in the respective figure legends). *P*-values of * < 0.05, ** < 0.01, and *** < 0.001 were considered statistically significant. Values higher than a *p*-value of 0.05 were regarded as not significant (ns).

## SUPPLEMENTARY MATERIALS FIGURES



## Abbreviations

ATF6: activating transcription factor 6
AV: annexin V
BZ: bortezomib
ER: endoplasmic reticulum
DMSO: dimethyl sulfoxide
ERAD: ER-associated protein degradation
FBS: fetal bovine serum
IRE1α: inositol-requiring enzyme 1α
MC: mast cell
MCL: mast cell leukemia
MM: multiple myeloma
PERK: double-stranded RNA-activated protein kinase-like ER kinase
PI: propidium iodide
RIDD: regulated IRE1-dependent decay of mRNA
SM: systemic mastocytosis
TKI: tyrosine kinase inhibitor
TM: tunicamycin
UPR: unfolded protein response
