# Supplementary material for: Infliction of proteotoxic stresses by impairment of the unfolded protein response or proteasomal inhibition as a therapeutic strategy for mast cell leukemia
Source: Oncotarget. 2017 Dec 17;9(3):2984–3000. doi: 10.18632/oncotarget.23354 (PMC5790440; doi:10.18632/oncotarget.23354)
Supplement: Supplementary file 1 [file oncotarget-09-2984-s001.pdf]

Infliction of proteotoxic stresses by impairment of the unfolded protein response or proteasomal inhibition as a therapeutic strategy for mast cell leukemia

SUPPLEMENTARY MATERIALS

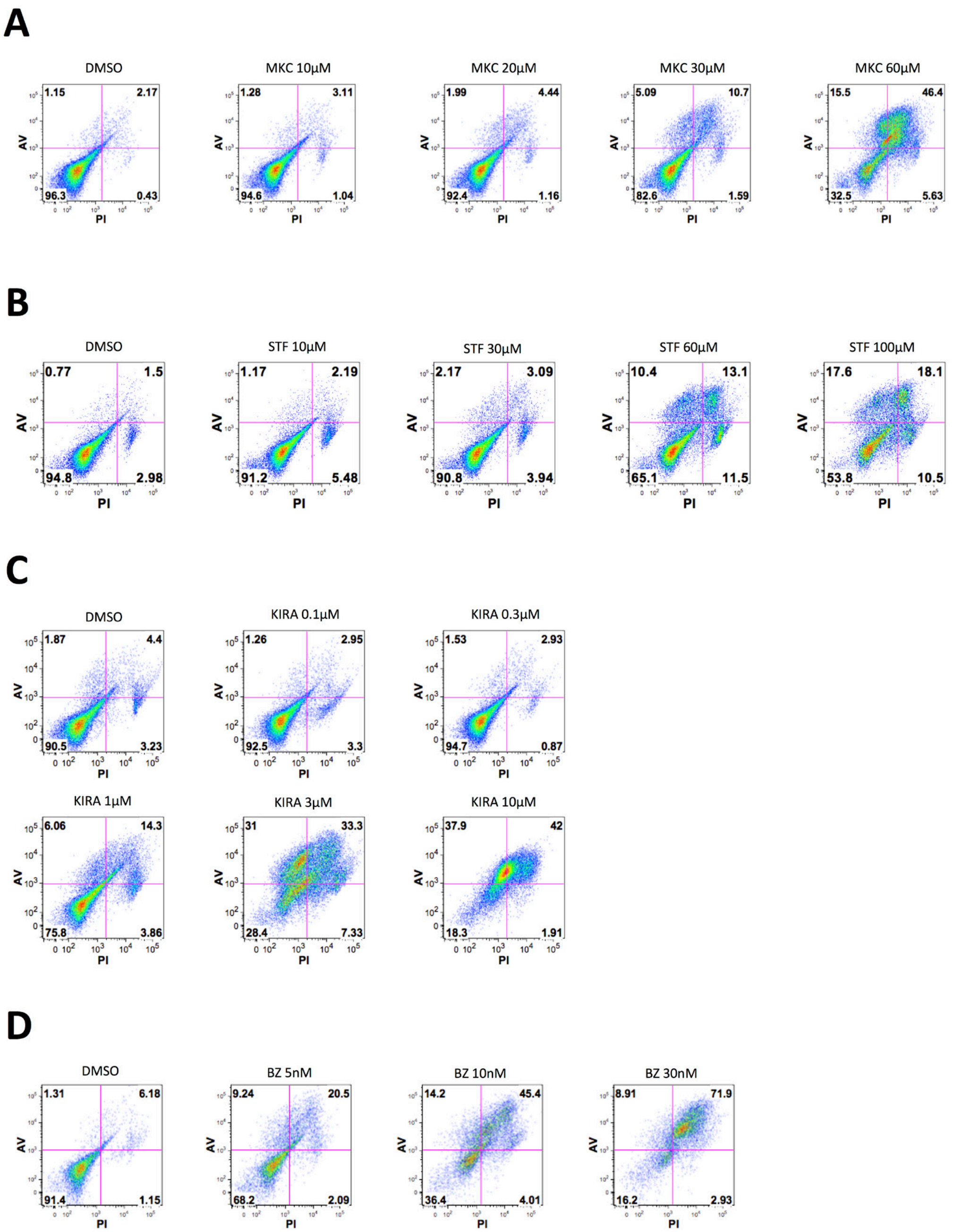

Supplementary Figure 1: Induction of cell death by MKC-8866, STF-083010, KIRA6, and BZ. HMC-1.2 cells were treated for 72h with the indicated substances/concentrations and cell viability was determined by FACS analysis of Annexin V (AV)/propidium iodide (PI) positivity. One representative experiment is shown.

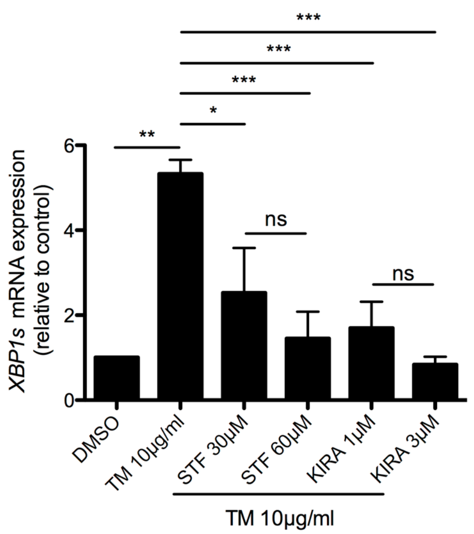

Supplementary Figure 2: STF and KIRA6 reduce TM-induced splicing of *XBP1*. HMC-1.2 cells were treated for 1h with vehicle (DMSO) or the indicated concentrations of STF or KIRA6 followed by a 6h treatment with 10µg/ml TM. *XBP1s* expression was evaluated by RT-qPCR and normalized to *HPRT*. (n=3)

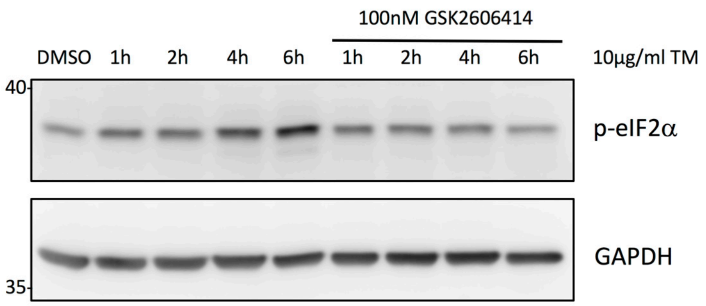

Supplementary Figure 3: GSK reduces TM-induced phosphorylation of eIF2α. HMC-1.2 cells were treated with vehicle (DMSO) or 100 nM GSK for 1h followed by incubation with 10µg/ml TM for the indicated time points and phosphorylation of eIF2α was detected by Western blotting. GAPDH served as loading control (n=2).
